# Low Testosterone and High Leptin Activate PPAR Signaling to Induce Adipogenesis and Promote Fat Deposition in Caponized Ganders

**DOI:** 10.3390/ijms25168686

**Published:** 2024-08-09

**Authors:** Mingming Lei, Yaxin Li, Jiaying Li, Jie Liu, Zichun Dai, Rong Chen, Huanxi Zhu

**Affiliations:** 1 Institute of Animal Science, Jiangsu Academy of Agricultural Sciences, Nanjing 210014, China; 20140036@jaas.ac.cn (M.L.); liyaxin6664@163.com (Y.L.); lijiaying915@163.com (J.L.); liujie891213@163.com (J.L.); 20210064@jaas.ac.cn (Z.D.); chenrong_big@163.com (R.C.); 2 Key Laboratory of Crop and Livestock Integration, Ministry of Agriculture, Institute of Animal Science, Jiangsu Academy of Agricultural Sciences, Jiangsu Province Engineering Research Center of Precision Animal Breeding, Nanjing 210014, China

**Keywords:** goose, abdominal fat, testosterone, leptin, PPARγ

## Abstract

Low or insufficient testosterone levels caused by caponization promote fat deposition in animals. However, the molecular mechanism of fat deposition in caponized animals remains unclear. This study aimed to investigate the metabolomics and transcriptomic profiles of adipose tissues and study the effect of testosterone and leptin on the proliferation of adipocytes. We observed a significant enlargement in the areas of adipocytes in the abdominal fat tissues in capon, as well as increased luciferase activity of the serum leptin and a sharp decrease in the serum testosterone in caponized gander. Metabolomics and transcriptomic results revealed differentially expressed genes and differentially expressed metabolites with enhanced PARR signal pathway. The mRNA levels of peroxisome proliferators-activated receptor γ, fatty acid synthase, and suppressor of cytokine signaling 3 in goose primary pre-adipocytes were significantly upregulated with high leptin treatment and decreased significantly with increasing testosterone dose. Hence, reduced testosterone and increased leptin levels after caponization possibly promoted adipocytes proliferation and abdominal fat deposition by altering the expression of PPAR pathway related genes in caponized ganders. This study provides a new direction for the mechanism through which testosterone regulates the biological function of leptin and fat deposition in male animals.

## 1. Introduction

Goose rearing is an important form of poultry production, and goose meat consumption is increasing in China and several European countries [1]. Goose capons are specially consumed in several parts of China for faster growing and better-quality meat than intact ganders [2]. Capon meat is more delicious than that of intact ganders because caponization can significantly improve intermuscular fat and reduce muscle cross-sectional area and muscle shear force [3].

Caponization can result in a deficiency of or reduction in testosterone in male animals. The low or insufficient testosterone levels increased the expression levels of the fatty acid synthesis-associated genes and promoted fat deposition in ganders and mice [4,5]. In addition to significantly reducing plasma testosterone levels in castrated rats, caponization can also increase plasma leptin (LEP) levels in castrated mice [3,5,6]. Although the goose leptin gene and protein have not been reported yet, it is confirmed that suppressor of cytokine signaling 3 (SOCS3) acts as a negative feedback factor for leptin signaling [7]. High levels of SOCS3 can reduce sensitivity of leptin, leading to leptin resistance and promoting fat deposition [8]. Our previous studies showed that the *SOCS3* mRNA expression was significantly upregulated in the abdominal fat (AF) and liver tissues of the caponized geese than those of the non-caponized geese [4]. These studies showed that testosterone and LEP possibly play an important role in fat deposition in animal capons. However, the relationship between testosterone and LEP in goose serum, as well as their regulatory mechanisms on male goose fat lipogenesis, are currently unclear.

In this study, we first investigated the AF percentage, the areas of adipocyte size in AF tissues, and the luciferase activity of the goose serum LEP in caponized geese and intact geese. On this basis, the global gene expression and metabolic profiles of the AF following caponization were performed using RNA-seq and liquid chromatography–tandem mass spectrometry (LC–MS/MS) in the Sanhua goose, a typical local breed in China. Moreover, we studied the effects of different doses of testosterone and LEP on the proliferation of goose primary pre-adipocytes. The goal was to clarify the effects of testosterone and LEP on regulating fat metabolism in ganders and to reveal the molecular mechanism of increased fat deposition in caponized geese.

## 2. Results

### 2.1. Low Testosterone Induces AF Deposition in Caponized Ganders

**Figure 1:**
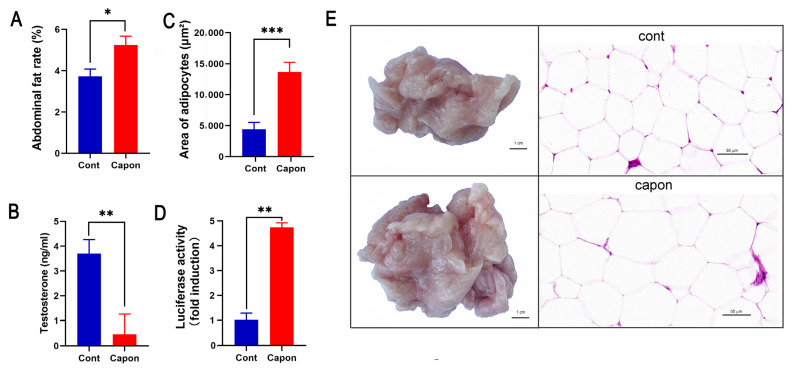
Changes of AF rate, area of adipocytes, testosterone, and LEP between caponized (Capon) and intact (Cont) geese at 45 days after caponization. (**A**) AF weight. (**B**) Percentage of AF. (**C**) Area of adipocytes on H&E-stained sections. (**D**) Luciferase activity of goose serum LEP in HEK-293 cell line stably expressing the chicken LEPR. (**E**) Representative images of AF tissues and H&E-stained sections of the tissues (×40). Data are means ± SD (n = 10); *, *p* < 0.05; **, *p* < 0.01, ***, *p* < 0.001.

The AF rates were higher in the capons than in the control sham-operated ganders (*p* < 0.05) at 45 days after caponization (Figure 1A). The area of the adipocytes (13,665.3 × 629.28 μm^2^) in the caponized group was significantly higher than that of the control group (4403.78 × 455.32 μm^2^) (*p* < 0.001) (Figure 1C), and the adipocytes in the AF tissues were evidently enlarged in the caponized group (Figure 1E). The serum testosterone of the caponized ganders decreased sharply (Figure 1B). The luciferase activity was only weakly induced by the serum of the control gander, while the serum of the caponized ganders significantly augmented the luciferase activity (*p* < 0.01) (Figure 1D).

### 2.2. Global Transcript Profiles in AF

**Figure 2:**
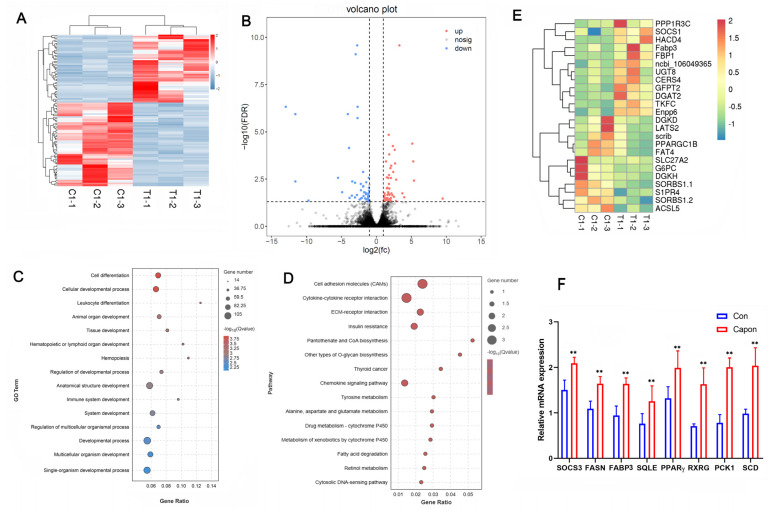
Effect of caponization on the global transcriptomic profiles of AF tissues. (**A**) Heatmap of samples from the control and caponized groups of geese. (**B**) Volcano plots showing significant DEGs in the AF of the control and caponized groups. Red and blue dots represent the upregulated and downregulated DEGs in caponization geese (n = 3), respectively. (**C**) The top 15 significantly enriched GO pathways for the DEGs. (**D**) The top 15 significantly enriched KEGG pathways for the DEGs. (**E**) Heatmap of the DEGs related to fat metabolism. (**F**) The expression of eight genes using RT-PCR. **, *p* < 0.01.

To investigate the effect of caponization on fat deposition, we examined the transcriptomes of AF tissues from caponized and intact ganders. Among the 16,091 detected transcripts, we identified 426 differentially expressed genes (DEGs) between the intact and caponized geese, including 263 upregulated and 173 downregulated genes. A heatmap of the two groups (Figure 2A) indicated good repeatability. Figure 2B shows a volcano plot of the DEGs, and Figure 2E shows the heatmap of 25 DEGs between the two groups. 

The functional categories were determined based on the gene annotations (Figure 2C). The most significantly enriched GO terms for the DEGs were cell differentiation, cell developmental process, leukocyte differentiation, animal organ development, and tissue development. The 15 most significantly enriched KEGG pathways for the DEGs were the cell adhesion molecules (CAMs), cytokine–cytokine receptor interaction, ECM–receptor interaction, insulin resistance, pantothenate and CoA biosynthesis, other types of O-glycan biosynthesis, chemokine signaling pathway, tyrosine metabolism, alanine, aspartate and glutamate metabolism, metabolism of xenobiotics by cytochrome P450, fatty acid degradation, retinol metabolism, and cytosolic DNA-sensing pathway (Figure 2D). We analyzed the expression of eight genes to confirm their expression profiles and validate the transcriptome data (Figure 2F), and the results confirmed that our data and the transcriptome sequencing platform were reliable. 

### 2.3. Global Metabolic Profiling of AF Tissues

**Figure 3:**
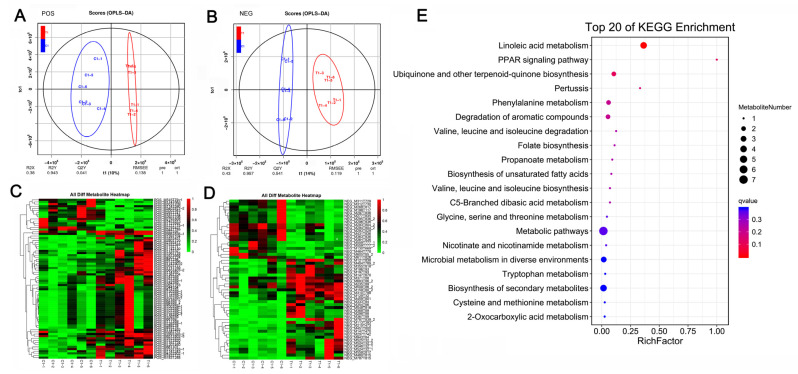
Effect of caponization on the global metabolomic profiles in AF tissues. POS, positive ion mode; NEG, negative ion mode. (**A**,**B**) OPLS-DA scores showing significant differences between the caponized and control groups. (**C**,**D**) Heatmap analysis of the DEMs between the two groups. (**E**) Significantly altered metabolic pathways. The size and color of each circle represent the pathway rich factor and *p*-value, respectively.

At 45 days after caponization, the global metabolomic profiles of the AF tissues were obtained from the control and caponized geese to further elucidate the effects of caponization on fat metabolites. We identified 75 (60 upregulated and 15 downregulated) differentially expressed metabolites (DEMs) and 54 (34 upregulated and 20 downregulated). DEMs, in positive and negative modes, respectively. OPLS-DA revealed significant differences between the groups in both positive and negative ion modes (Figure 3A,B). Heatmaps of the 129 DEMs identified in positive and negative modes are shown in Figure 3C,D, respectively. Figure 3E shows the top 20 KEGG enrichment pathways for the DEMs. Among these pathways, the most important are the linoleic acid pathway, PARR pathway, and the biosynthesis of unsaturated fatty acids. 

### 2.4. Integrated Analyses of the Metabolomic and Transcriptomic Data

**Figure 4:**
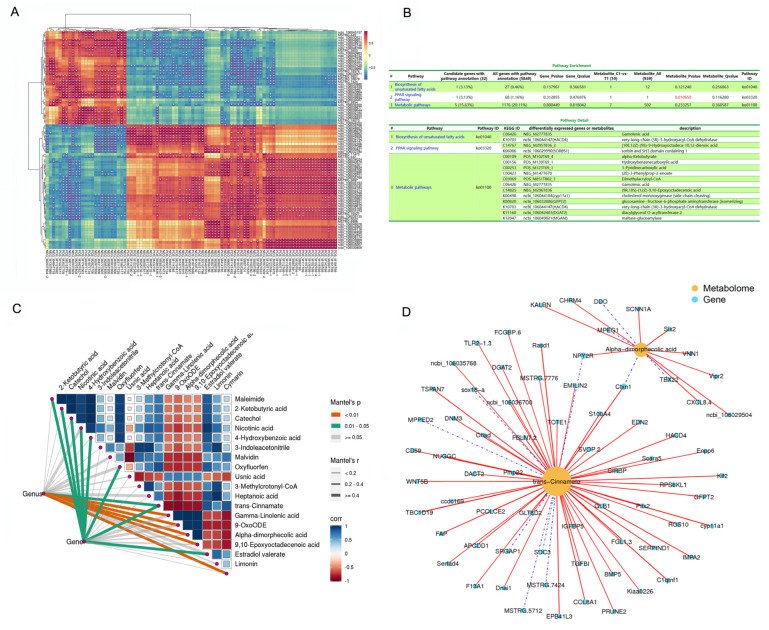
Integrated enrichment analysis of DEMs and DEGs. (**A**) Heatmap of the DEMs and DEGs. Gene names are shown in italics, and metabolite names are shown in normal font. The color of the squares in the graph represents correlation, with darker colors indicating stronger correlation. *, *p* < 0.05. (**B**) KEGG pathway enrichment of DEMs and DEGs and pathway detail. (**C**) Variations in metabolites that have an obvious relationship with the DEGs in AF tissue. Metabolome composition and fat metabolism gene expression in both groups of geese (derived from AF metabolome and RNA-Seq transcriptomic data, respectively) that were related to the 18 significant DEMs using Mantel test. Edge width corresponds to the Mantel’s r statistic for the corresponding distance correlations, and edge color indicates the statistical significance. The color gradient indicates the Pearson’s correlation coefficient. (**D**) DEGs related to trans-cinnamate and alpha-dimorphic acid. The metabolites are shown as yellow circles. Blue squares represent genes.

The heatmap showed significant correlations between the levels of several DEMs and DEGs (*p* < 0.05; Figure 4A), including (10E,12Z)-(9S)-9-Hydroxyoctadeca-10,12-dienoic acid (NEG_M295T836_2), *Insulin-like growth factor binding protein 5 (IGFBP5), interferon regulatory factor 4* (*IRF4*), *ectonucleotide pyrophosphatase/phosphodiesterase 6* (*ENPP6*), *t-complex-associated-testis-expressed 1* (*TCTE1*), *glutamine fructose-6-phosphate transaminase 2* (*GFPT2*), *transforming growth factor beta induced protein* (*TGFBI*), *bone morphogenetic protein 5* (*BMP5*), *3-hydroxyacyl-CoA dehydratase 4* (*HACD4*), *metallophosphoesterase domain containing 2* (*MPPED2*), *and maltase-glucoamylase* (*MGAM*). The KEGG of the DEGs and DEMs is significantly enriched in the PPAR signaling pathway.

The Mantel test results corroborated the correlations among the data (Figure 4C). Variations in metabolites were notably associated with the expression levels of the DEGs in AF tissue. The expression of metabolism-related genes in both groups of geese was related to each of the 18 significant DEMs, as revealed by Mantel test. Moreover, the levels of 2-ketobutyric acid, catechol, nicotinic acid, 4-hydroxybenzoic acid, oxyfluorfen, trans-cinnamate, and estradiol valerate were highly correlated (Mantel’s r > 0.4, 0.01 < *p* < 0.05; Figure 4C). Trans-cinnamate and alpha-dimorphecolic acid, which are involved in fat metabolism and lipid droplet aggregation, were correlated with all related DEGs (Figure 4D), including *dexamethasone-induced Ras-related protein 1* (*Rasd1*), *cytochrome P450 family member 11A1* (*CYP11a1*), *paired-like homeodomain transcription factor 2* (*Pitx2*), *C1q and tumor necrosis factor related protein 1* (*C1QTNF1*), *cortexin- 1* (*CTXN1*), *erythrocyte membrane protein band 4.1 like 3* (*EPB41L3*)*, IGFBP5, BMP5, ENPP6, triacylglycerol acyltransferase 2* (*DGAT2*), and *peripheral myelin Protein 22* (*PmP22*).

### 2.5. PPARγ Signaling Pathway Mediated LEP- and Testosterone-Induced Adipogenesis

**Figure 5:**
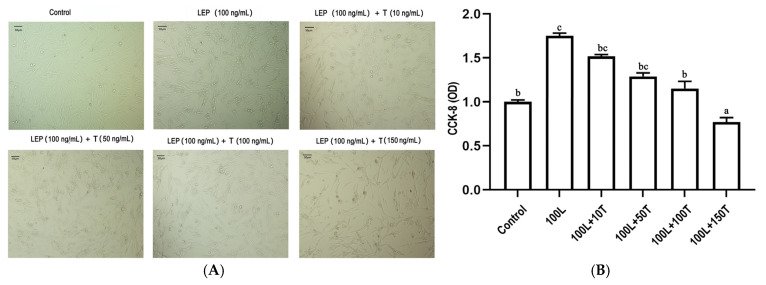
The effect of LEP and testosterone on proliferation of goose primary pre-adipocytes. (**A**) adipocytes were treated with 100 ng/mL of LEP or 10, 50, 100, and 150 ng/·mL of testosterone for 24 h. (**B**) cell viability was assayed by CCK-8. Different letters above the bars denote significant differences (a,b: *p* < 0.05; b,c: *p* < 0.05; a–c: *p* < 0.01).

To investigate the effects of LEP and testosterone on goose primary pre-adipocyte proliferation, a cell-counting kit-8 (CCK-8) assay was performed to quantify cell viability after 24 h treatment with LEP (100 ng/mL) and increasing concentrations of testosterone (10, 50, 100, and 150 ng/mL). As shown in Figure 5, LEP (100 ng/mL) increased cell viability and testosterone significantly decreased cell viability in a dose-dependent manner.

**Figure 6:**
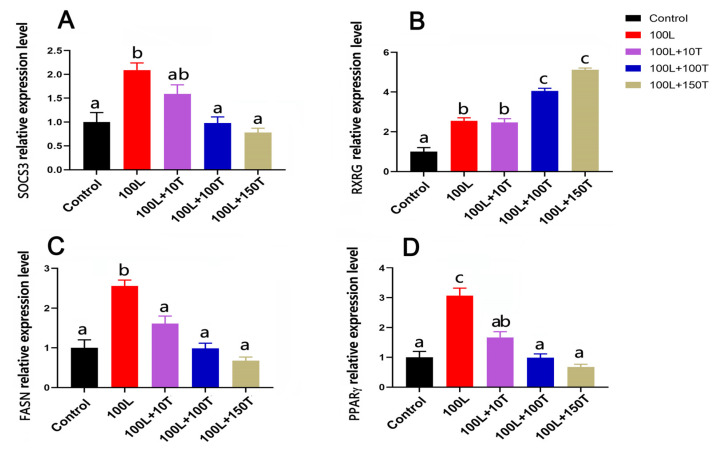
The mRNA expressions of the four genes of adipocytes treated with LEP and testosterone. *SOCS3* (**A**), *RXRG* (**B**), *FASN* (**C**), and *PPARγ* (**D**) mRNA levels were examined in pre-adipocytes with LEP (L; 100 ng/mL) with and without testosterone (T; 10, 100, and 150 ng/mL) treatment for 24 h. Different letters above the bars denote significant differences (a,b: *p* < 0.05; b,c: *p* < 0.05; a–c: *p* < 0.01).

The *SOCS3*, *peroxisome proliferator activated receptor γ* (*PPARγ)*, and *fatty acid synthase* (*FASN*) genes’ mRNA levels were significantly upregulated after treatment with 100 ng/mL LEP, and this effect was gradually reduced with the addition of increasing doses of testosterone (Figure 6). In the presence of 100 ng/mL testosterone, the mRNA levels of *FASN* and *PPARγ* significantly decreased, and this effect was maintained at 150 ng/mL testosterone. After treatment with 100 ng/mL LEP, the level of *retinoid X receptor gamma* (*RXRG*) mRNA increased. When LEP (100 ng/mL) and testosterone (10 ng/mL) were added simultaneously, there was no significant change in the expression of *RXRG* in adipocytes. However, when testosterone concentration was 100 or 150 ng/mL, the expression of *RXRG* in adipocytes was significantly upregulated. 

## 3. Discussion

Testosterone is an important androgen that negatively regulates body fat deposition in male animals [9,10]. Removing the testicles of male livestock to decrease testosterone levels and increase body fat content is an important method to improve meat quality. In this study, we investigated the metabolomic and transcriptomic profiles of AF in caponized geese at 45 d after testicular resection and found that low testosterone and high LEP caused by caponization promoted the expression of genes related to lipogenesis through PPAR signaling, thereby promoting fat deposition. High LEP levels in goose primary pre-adipocytes increased *SOCS3* mRNA expression, while high testosterone levels decreased it. Therefore, low testosterone and high LEP levels jointly promote fat deposition in caponized geese.

Firstly, I would like to demonstrate the effectiveness of detecting serum LEP concentration in geese. Hen (2008) reported on the construction of a LEP bioassay based on the activation of chicken LEP receptor in a 293 cultured cell line and found that human and cow serum could activate fluorescence signals [11]. Subsequently, chicken and duck LEPs directed the production of secreted proteins with LEP activity and could also activate fluorescence signals of the cLEPR expression system [12]. Moreover, we obtained the LEP gene sequence of goose, which shared 84% homology with the LEP sequence of ducks, and the goose LEP proteins expressed in *Pichia pastoris* were found to activate the fluorescence signals of the cLEPR expression system (unpublished data). Waterfowl, including duck and goose, have a relatively higher fat content compared to chicken. So, the activation of fluorescent signals by goose serum LEP is reliable and effective.

An important change caused by caponization is the significant decrease in serum testosterone levels. Caponization significantly reduced testosterone levels and increased subcutaneous, intercellular, and AF content compared to the fat accumulation in intact cockerels [13,14]. Low testosterone levels in caponized geese reportedly increase fat deposition, leading to weight gain [4]. Our histological analysis showed that the adipocytes in the AF tissues of caponized geese were evidently enlarged. This result was confirmed that weight gain in rats fed a high-fat diet was shown to be due to a significant increase in the large adipocytes [15]. In castrated mice, hypogonadism increased obesogenic fat mass expansion through adipocyte hypertrophy and adipogenesis, and high testosterone levels effectively reduced HFD-induced fat mass expansion [16,17]. Testosterone-treated cultured brown adipocytes exhibited fewer and smaller lipid droplets than control cells [18]. These results indicated that the increases in adipocyte size are important causes of the increase in AF weight in caponized geese.

Another important change caused by caponization is a significant increase in LEP levels. In the present study, the luciferase activity was only weakly induced by the serum of the control ganders, while the serum of the caponized ganders significantly augmented the luciferase activity. The stronger the fluorescence signal, the higher the concentration of LEP. The results showed that LEP levels in the serum of caponized geese were higher than in the control geese. LEP, a peptide secreted by adipocytes, is an important indicator of fat content in animals [19,20]. The significant increase in LEP levels in our study also indicated an increase in fat deposition in the caponized geese. Similar findings have been previously reported, including the following: the highest LEP levels were observed in animals with testosterone deficiency induced by caponization [5,21]. Using goose pre-adipocytes, we showed that *SOCS3* and *FASN* mRNA levels were significantly higher in cells treated with 100 ng/mL than in those treated with 10 ng/mL LEP. This result is consistent with previous findings [4]. Excessive LEP can lead to LEP resistance [22], which promotes the differentiation of precursor adipocytes and the formation of lipid droplets.

In this present study, integrated analyses of metabolomics and transcriptomic data showed KEGG enrichment of DEMs and DEGs in the PPAR signaling pathway. The PPAR signaling pathway is the most important pathway involved in tissue-specific lipid deposition [23,24,25,26]. Our results suggest that caponization promotes fat deposition in geese by activating the PPAR signaling pathway. Six representative genes were significantly upregulated (*apolipoprotein A-1 (APOA1), stearoyl CoA desaturase (SCD1)*, *fatty acid binding protein 7 (FABP7)*, *RXRG*, and *fatty acid desaturase (FADS2)*) or downregulated *(fatty acid binding protein 3*), and these genes were strongly associated with the PPAR pathway [27,28]. Consistent with this, Cui et al. (2018) reported that the caponization of male chickens accelerates AF deposition by altering the expression of related genes through the PPAR pathway [13].

LEP and PPARs are derived from adipose tissues and play important roles in the regulation of lipid metabolism [29]. Various studies have shown crosstalk between PPARs and LEP and the effect of PPARγ agonists on LEP expression and signaling [30,31]. Activation of central PPARγ promoted food intake and body weight gain [32]; moreover, preventing the increase in peroxisome density within POMC neurons through selective deletion of PPARγ elevated the ROS levels with concomitant increases in POMC activity and LEP sensitivity [31]. Hypothalamic *PPARγ* mRNA expression was several folds higher in mice with diet-induced obesity than in the lean controls [30]. In the present study, high LEP increased the mRNA levels of *PPARγ* and *SOCS3* in goose adipocytes. This suggests that obesity caused by caponization is closely related to LEP resistance, similar to a previous finding that exogenously administered LEP did not induce a signal transducer and activator of transcription 3 (STAT3) phosphorylation and or increase SOCS3 [33,34]. In our study, as the dose of testosterone increased, *SOCS3* mRNA levels decreased, suggesting that testosterone may regulate transduction of the LEP signal through SOCS3. The above results will help us to understand the relationship between LEP resistance and low testosterone levels in obese men and help them to lose weight based on the relationship.

## 4. Materials and Methods

### 4.1. Animals and Experimental Design

Animal experiments were performed as previously described [4]. In brief, 15 sham-operated geese (the control group) and 15 caponized geese were selected at 150 days of age and reared until 195 days of age. These geese were raised using the conventional method of stocking and supplementary feeding (11.69 MJ/kg ME, 12.5% CP, 0.11% calcium, and 0.14% available phosphorus). In addition to the feed, the geese were free to eat grass during grazing. The geese were maintained under natural daylight and temperatures. After 45 days of caponization, 10 geese were randomly selected from each group to be slaughtered. They were electrically stunned (120 V/50 Hz for 5 s) and exsanguinated by severing the jugular vein and carotid artery on one side of the neck. Afterward, they were passed through a warm scalding vessel (60 °C for 2 min) and a plucker (2 min) and were then manually eviscerated. The tissue samples of the AF were collected. One portion of the AF tissue was preserved in 4% paraformaldehyde fixative for histopathological examination, and the other portion was snap-frozen in liquid nitrogen for non-targeted metabolomics and RNA sequencing analyses. 

### 4.2. Serum LEP Bioassay

LEP activity was measured using a bioassay consisting of HEK-293 cells expressing exogenous full-length chicken LEPR cDNA and the firefly luciferase gene, under the control of a STAT3 binding element as described before [10,35]. The cells were incubated under a humidified atmosphere containing 5% CO_2_ at 37 °C for 24 h, and then washed with PBS to remove any unattached cells. The following day, the medium was replaced with fresh DMEM (300 μL) or with medium containing goose serum samples (30 μL/well), in quadruplicate. After a 4 h incubation, the medium was aspirated, and the cells were lysed by the addition of 100 μL Promega cell lysis reagent after 4 h. Luciferase activity was measured using the TD20e luminometer (Turner Design, Mountain View, CA, USA). Fold induction of the reporter gene was calculated by dividing luciferase activity of the control treated and untreated cells in each plate. 

### 4.3. Hematoxylin–Eosin Staining Evaluation

Hematoxylin and eosin staining of AF tissue was performed. Briefly, adipose tissues were washed thrice with cold saline solution, fixed with 4% paraformaldehyde, embedded in paraffin, cut into 4 μm sections, and then stained with H&E. Histological changes in adipose tissues, including the area of adipocytes and the number of adipocytes per unit area, were evaluated using a Pannoramic DESK scanner (3DHISTECH Ltd., Budapest, Hungary).

### 4.4. mRNA-Sequencing

To determine the mRNA expression profiles in the AF tissue of caponized and intact geese, tissue samples from intact caponized ganders were sent to GeneDenovo Biotechnology Co. (Guangzhou, China) for mRNA extraction, quality control, library construction, and sequencing. Clean reads were mapped to the reference Anser cygnoides genome using HISAT2.2.4 and assembled using StringTie v1.3. 

The differentially expressed genes (DEGs) were obtained by comparing the mRNA expression profiles of the two goose groups using DESeq2_1.44.0 software. Genes with a false discovery rate (FDR) < 0.05 and an absolute fold change > 1.5 were considered DEGs. Gene Ontology (GO) terms and Kyoto Encyclopedia of Genes and Genomes (KEGG) pathways with an FDR < 0.05 were considered significantly enriched (http://www.genome.jp/tools/kaas/, accessed on 6 July 2023)

### 4.5. Metabolomics Analysis

Untargeted metabolomics was analyzed by GeneDenovo Biotechnology Co. (Guangzhou, China). First, 12 randomly selected AF tissue samples (approximately 25 mg) from the two groups were homogenized and sonicated in an ice-water bath. After incubation at −20 °C for 1 h, the samples were centrifuged at 12,000× *g* for 15 min at 4 °C. The supernatants were analyzed through LC–MS/MS using a Vanquish UHPLC system with an Orbitrap MS UPLC HSS T3 column (Thermo Fisher Scientific, Waltham, MA, USA).

All raw GC–MS data were subjected to batch molecular feature extraction using MassHunter_B.08 (Agilent, Santa Clara, CA, USA). Unsupervised PCA and supervised OPLS-DA analyses were performed using SIMCA-P software (version 13.0; Umetrics, Umeå, Sweden) to identify differential metabolites between the two groups. All variables were Pareto-scaled prior to analysis. A variable importance in projection (VIP) value > 1.0 and *p* < 0.05 were set as the statistical threshold for discriminating significant differential metabolites.

### 4.6. Integrative Analysis of Transcriptome and Metabolome Data

We mapped DEGs in the transcriptome and DEMs in the metabolome to the KEGG pathway and selected DEGs and DEMs in the shared pathway for analysis. Based on the KEGG enrichment pathway, heat maps were used to show the potential relationship between genes and metabolite expression in the CG, CS, and CT group pathways. Finally, the information network of DEGs and DEMs enriched in the main pathways was mapped by using the cell landscape to reveal the changes of genes and metabolites caused by low temperature stress.

### 4.7. Isolation of Goose Pre-Adipocytes and Cell Culture

Goose primary pre-adipocytes were isolated from the AF tissues of a 20-day-old Sanhua goose as previously described [36]. Briefly, goose AF tissues were collected and washed with PBS supplemented with penicillin (100 U/mL) and streptomycin to remove all visible connective tissues. The washed AF tissues were minced into 1 mm^2^ pieces using scissors, and a portion (1.5–2.0 mL) was digested with collagenase I (0.2 U/L) in 15 mL of Hank’s Balanced Salt Solution (HBSS; Gibco, Grand Island, NY, USA) for 45–60 min at 37 °C. After incubation, Dulbecco’s modified Eagle’s medium containing 10% FBS and 100 U/mL penicillin and streptomycin was added to stop digestion. The mixture was passed through sterile 0.0750 mm and 0.0374 mm nylon mesh filters. The filtered suspensions were centrifuged at 1000× *g* for 10 min, and the precipitates were resuspended in 1 mL of Blood Cell Lysis Buffer (Invitrogen) and incubated at 25 °C for 15 min. The cells were rinsed with growth medium and centrifuged twice at 1000× *g* for 5 min. Viable cells were counted using 0.4% Trypan blue and a hemocytometer. Finally, the obtained pre-adipocytes were resuspended in the aforementioned growth medium, seeded into culture flasks at 1 × 10^6^ cells/mL, and cultured in a humidified atmosphere of 5% CO_2_ and 95% air at 37 °C. 

After 12 h of culture, pre-adipocytes were treated with the human LEP protein (Abcam, Cambridge, UK). The LEP (100 ng/mL) were added to the medium, or combined with different doses of testosterone (10, 50, 100, and 150 ng/mL). For 24 h later, cell viability was performed using a CCK-8 assay. Adipocytes were collected for qPCR.

### 4.8. Quantitative RT-PCR

Total RNA from the AF and goose primary adipocytes was extracted with TRIzol reagent using a commercial kit (Invitrogen, Carlsbad, CA, USA) according to the manufacturer’s instructions. Gene-specific primers were designed using Primer 3.0 software (www.ncbi.nlm.nih.gov/tools/primer-blast/, accessed on 11 August 2023). based on sequences in GenBank. Amplified products were detected using an ABI PRISM_7500 (Applied Biosystems, Foster City, CA, USA). The 2^−ΔΔCT^ method was used to determine relative mRNA expression, with *β*-actin as a control.

### 4.9. Statistical Analysis

All data are expressed as mean ± SEM. Differences in AF rate, testosterone concentration, the area of adipocytes, and gene expression of abdominal fat tissues were analyzed by one-way ANOVA in the animal experiment with surgical testicular removal as a factor using SPSS 20 (SPSS Inc., Chicago, IL, USA). The same method was applied to analyze LEP luciferase activity by goose serum LEP. Differences in cell viability and gene expression levels of goose primary adipocytes were analyzed by two-way ANOVA with different dose of leptin and testosterone as factors using SPSS 20 (SPSS Inc., Chicago, IL, USA). Statistical significance was set at *p* < 0.05. All images were drawn using GraphPad Prism V8.0 (GraphPad Software, San Diego, CA, USA).

## 5. Conclusions

In conclusion, our study suggested that reduced testosterone and increased LEP levels after caponization enlarges the size of adipocytes and accelerates AF deposition by changing the expression of related genes through PPAR pathways in the AF of ganders. Moreover, testosterone may regulate transduction of the LEP signal through SOCS3, thereby affecting the proliferation of adipocytes. This study provides important new insights into the mechanism through which testosterone regulates the biological function of LEP and fat deposition in male animals. However, the detailed molecular mechanism through which testosterone regulates LEP through SOCS3 remains to be elucidated.

## Data Availability

The raw data supporting the conclusions of this article will be made available by the authors on request.
